# Culprits for Retrosternal Deformation After Coronary Artery Bypass Surgery

**DOI:** 10.21470/1678-9741-2021-0507

**Published:** 2022

**Authors:** Abdulkerim Özhan, Murat Baştopçu

**Affiliations:** 1 Cardiovascular Surgery, Göztepe Prof. Dr. Süleyman Yalçın City Hospital, Istanbul, Turkey.; 2 Cardiovascular Surgery, Tatvan State Hospital, Bitlis, Turkey.

Dear Editor,

We have read with interest the article “Retrosternal Deformations after Coronary Artery Bypass Surgery Using Statistical Shape Analysis” by Bademci et al.^[1]^ The authors have used novel statistical methods to demonstrate geometric changes in the mediastinum following coronary artery bypass grafting (CABG) surgery.

The authors conclude that, following CABG, “the main pulmonary artery approximates to the sternum” with “narrowing of the retrosternal area”. They attribute this finding to the formation of scar tissue and adhesions which are the result of inflammation associated with cardiac surgery. We would like to inquire if the authors believe operative techniques affect the geometric changes in the mediastinum. Surgeons vary greatly in their CABG techniques, choice of grafts, pericardial closure, and use of cardiopulmonary bypass (CPB). On-pump CABG may precipitate inflammatory processes more potently compared to off-pump CABG^[2]^. Another technique that produces less inflammation is the mini-extracorporeal circulation (MECC) during CABG^[3]^. Therefore, postoperative adhesions and scar tissue formation could be attenuated in patients who are revascularized with off-pump CABG or using MECC.

It is also possible that CPB times, blood transfusions, perioperative medications, and patient factors affect the amount of inflammatory response in the postoperative period. Data related to CPB and other factors that induce inflammation in the patient group in the study may be relevant. The cannulation strategy is also important, as central arterial cannulation may result in more retrosternal adhesions than femoral cannulation. The placement of a pulmonary vent catheter and the extent of tissue dissection around the pulmonary artery may influence the adhesions present in the postoperative period.

For their statistical shape analysis, the authors have compared patients with previous CABG with a control group without a history of cardiac surgery. In our opinion, this overlooks interpersonal geometric differences in mediastinal anatomy. The size of the great vessels can be different in the population of patients who require cardiothoracic surgery than individuals who did not require an operation. Comorbidities accompanying heart diseases, including, but not limited to, aortic enlargement or elevated pulmonary pressure increasing great vessel diameters, may complicate the comparison of retrosternal distance^[4]^. If the authors can identify a cohort of patients who had chest computed tomography before and after surgery, their methods can be applied to distinctly assess the effect of CABG surgery on retrosternal adhesions. This would also allow controlling or comparing other important factors in inflammation.

We congratulate the authors for their study in the underexplored field of retrosternal adhesions in cardiac surgery. The findings from this study can guide cardiac surgeons who face the perils of retrosternal adhesions in redo operations.
